# A critical role for long-term potentiation mechanisms in the maintenance of object recognition memory in perirhinal cortex revealed by the infusion of zeta inhibitory pseudosubstrate

**DOI:** 10.3389/fnbeh.2022.970291

**Published:** 2022-10-03

**Authors:** Alexandra R. Outram, Malcolm W. Brown, Elizabeth Clea Warburton, Gareth R. I. Barker

**Affiliations:** School of Physiology, Pharmacology and Neuroscience, University of Bristol, Bristol, United Kingdom

**Keywords:** novel object recognition memory, perirhinal cortex, long-term potentiation, ZIP, rat

## Abstract

Object recognition, the ability to discriminate between a novel and a familiar stimulus, is critically dependent upon the perirhinal cortex. Neural response reductions upon repetition of a stimulus, have been hypothesized to be the mechanism within perirhinal cortex that supports recognition memory function. Thus, investigations into the mechanisms of long-term depression (LTD) in perirhinal cortex has provided insight into the mechanism of object recognition memory formation, but the contribution of long-term potentiation (LTP) to object recognition memory formation has been less studied. Inhibition of atypical PKC activity by Zeta Inhibitory Pseudosubstrate (ZIP) impairs the maintenance of LTP but not LTD, thus here infusion of ZIP into the perirhinal cortex allowed us to investigate the contribution of LTP-like mechanisms to object recognition memory maintenance. Infusion of ZIP into the perirhinal cortex of rats 24 h after the sample phase impaired performance in an object recognition but not an object location task, in contrast infusion of ZIP into the hippocampus impaired performance in an object location but not an object recognition task. The impairment in object recognition by ZIP was prevented by administration of the peptide GluA2_3y_, which blocks the endocytosis of GluA2 containing AMPA receptors. Finally, performance in a perceptual oddity task, which requires perirhinal cortex function, was not disrupted by ZIP. Together these results demonstrate the importance of LTP-like mechanisms to the maintenance of object recognition memory in the perirhinal cortex.

## Introduction

The perirhinal cortex (PRH) is necessary for object recognition memory and is also a storage site for such memory [for review Warburton and Brown (2010); Brown et al. (2012), and Brown and Banks (2015)]. Critically, PRH interventions that target plasticity mechanisms while leaving neurotransmission intact also impair single item object recognition memory (Warburton et al., 2005; Griffiths et al., 2008; Tinsley et al., 2012). To date, the evidence strongly indicates the involvement of plasticity mechanisms that result in synaptic weakening and have parallels in processes underlying long-term depression (LTD) (Warburton et al., 2003; Griffiths et al., 2008). A process involving synaptic weakening readily explains the observed reduction in neuronal responses observed in monkeys and rats when novel stimuli are seen again (Zhu et al., 1995; Xiang and Brown, 1998) and is consistent with computational modeling predictions that efficient storage in a familiarity discrimination network is only achievable if the learning algorithm includes a term producing synaptic weakening (Bogacz and Brown, 2003). However, an efficient network must maintain a balance of synaptic excitability to avoid either over- or under-reactivity. The question therefore arises as to whether recognition memory also relies on synaptic strengthening within PRH and, if so, does this strengthening process employ long-term potentiation-like mechanisms (LTP).

The atypical protein kinase C isoforms (protein kinase Mζ and ι/λ isoforms) are necessary for the maintenance of LTP but not LTD in the PRH and hippocampus (HPC) (Ling et al., 2002; Sajikumar et al., 2005; Serrano et al., 2005; Panaccione et al., 2013). Application of Zeta Inhibitory Pseudosubstrate (ZIP), an inhibitor of protein kinase Mζ (PKMζ) reversed a previously established LTP (Sajikumar et al., 2005) but not LTD and erased a spatial memory (Pastalkova et al., 2006). While the specificity of ZIP for PKMζ has been questioned (Wu-Zhang et al., 2012; Lee et al., 2013; Volk et al., 2013; LeBlancq et al., 2016) and at higher doses ZIP can act on PKCλ (Ren et al., 2013), the ability of ZIP to impair LTP but not LTD has not been disputed and the effect of ZIP administration on PRH memory function has not been studied. Atypical PKCs are thought to maintain memories by sustaining enhanced AMPA receptor levels in the post-synaptic density *via* an interaction with the GluA2 subunit of the AMPA receptor. The synthetic peptide GluA2_3y_ mimics the carboxy tail of the AMPA receptor and inhibits GluA2 receptor endocytosis. Thus, infusion of GluA2_3y_ prevented the amnesic effects of ZIP infusion in the HPC (Migues et al., 2010), amygdala (Migues et al., 2010), and medial prefrontal cortex (Evuarherhe et al., 2014).

The present study tested the following hypotheses 1. That ZIP infusion into PRH impairs the maintenance of single item novel object recognition but not object location memory 2. As object location but no single item novel object recognition depends on the HPC, we predict the reverse to be true in HPC. 3. ZIP infusion does not alter the perceptual functions of PRH. 4. That the memory impairment produced by ZIP infusion can be blocked by preventing GluA2 receptor endocytosis by infusion of the synthetic peptide GluA2_3y_.

## Methods

### Subjects

All experiments were conducted on adult male Dark Agouti rats (Bantin and Kingman, Hull, United Kingdom) weighing 230–250 g at the commencement of experiments. Animals were housed in pairs under a 12 h light/dark cycle (light phase, 20.00–08.00.). Behavioral training and testing were conducted during the dark phase of the cycle. Food and water were available *ad libitum*. All animal procedures were performed in accordance with United Kingdom Animals Scientific Procedures Act (1986). All efforts were made to minimize the suffering and the number of animals used.

Four cohorts of rats were used in this study. Cohort 1 consisted of 12 animals and was used to test the effect of ZIP infusion into PRH on the maintenance of object recognition and object location memory, two animals were lost from this cohort due to cannula blockages. Cohort 2 consisted of 10 animals and was used to test the effect of ZIP infusion into HPC on the maintenance of object recognition and object location memory, one animal was lost due to a blocked cannula. These animals had previously received infusion of D-AP5, data reported in Barker and Warburton (2015). Cohort 3 consisted of 13 animals and was used to test the effect of ZIP infusion into PRH on perceptual function, one animal was lost due a blocked cannula. Cohort 4 consisted of 12 animals and was used to test the effect of GluA2_3y_ infusion on ZIP-induced memory impairments, two animals were lost from this cohort due to blocked cannula.

### Cannula implantation

Implantation of cannulae followed previously described procedures (Warburton et al., 2003; Barker and Warburton, 2015). Briefly each rat was anesthetized with isoflurane (induction 4%, maintenance 2–3%) and secured in a stereotaxic frame with the incisor bar set to achieve flat skull. Stainless steel guide cannulae (26 gauge, Plastics One, Bilaney, Sevenoaks, United Kingdom) were implanted through burr holes in the skull at the following coordinates relative to bregma: HPC AP −4.8 mm, ML ± 2.6 mm, DV −3.0 mm from dura matter; PRH AP −5.6 mm, ML ± 4.5 mm, DV −6.7 mm from skull surface at 20° to vertical. All cannulae were anchored to the skull by stainless steel screws (Plastics One, Bilaney, United Kingdom) and dental acrylic. Following surgery, each animal received fluid replacement (5 mL saline, s.c.) and analgesia (0.05 mL Temgesic, i.m.) and then housed individually for 1-week post-surgery and then in pairs. Between infusions, 33-gauge obturators (Plastics One, Bilaney, United Kingdom) kept the cannulae patent.

### Intracerebral infusions

The selective PKMzeta inhibitor, ZIP (Tocris, Bristol, United Kingdom) or a scrambled ZIP peptide control (sZIP) (Tocris, Bristol, United Kingdom) were dissolved to a concentration of 10 mM (Pastalkova et al., 2006; Serrano et al., 2008) in physiological saline. The inhibitor of activity dependent endocytosis of GluA2 (GluA2_3y_) was conjugated to the HIV viral transduction domain (TAT) to allow the peptide to penetrate neuronal cell membranes. TAT-GluA2_3y_ (Anaspec, Fremont, USA) or scrambled TAT-GluA2_3y_ (Anaspec, United States) were dissolved to a concentration of 30 μM (Migues et al., 2010) in physiological saline. Infusions followed previously described procedures (Warburton et al., 2003), briefly, infusions were made through a 33-gauge infusion needle (Plastics One, Bilaney, United Kingdom) inserted into the implanted cannulae and attached to a 25-μL Hamilton syringe via polyethylene tubing. Drugs were infused into the HPC at a rate of 0.25 μl min^–1^ and into the PRH at a rate of 0.5 μl min^–1^ over a period of 2 min. The volumes have been used extensively previously (Winters and Bussey, 2005; Akirav and Maroun, 2006; Barker and Warburton, 2008) and have been shown to achieve a drug spread of 1–1.5 mm^3^ (Martin, 1991; Attwell et al., 2001). Following the infusion, the needle remained in place for a further 5 min.

**FIGURE 1:**
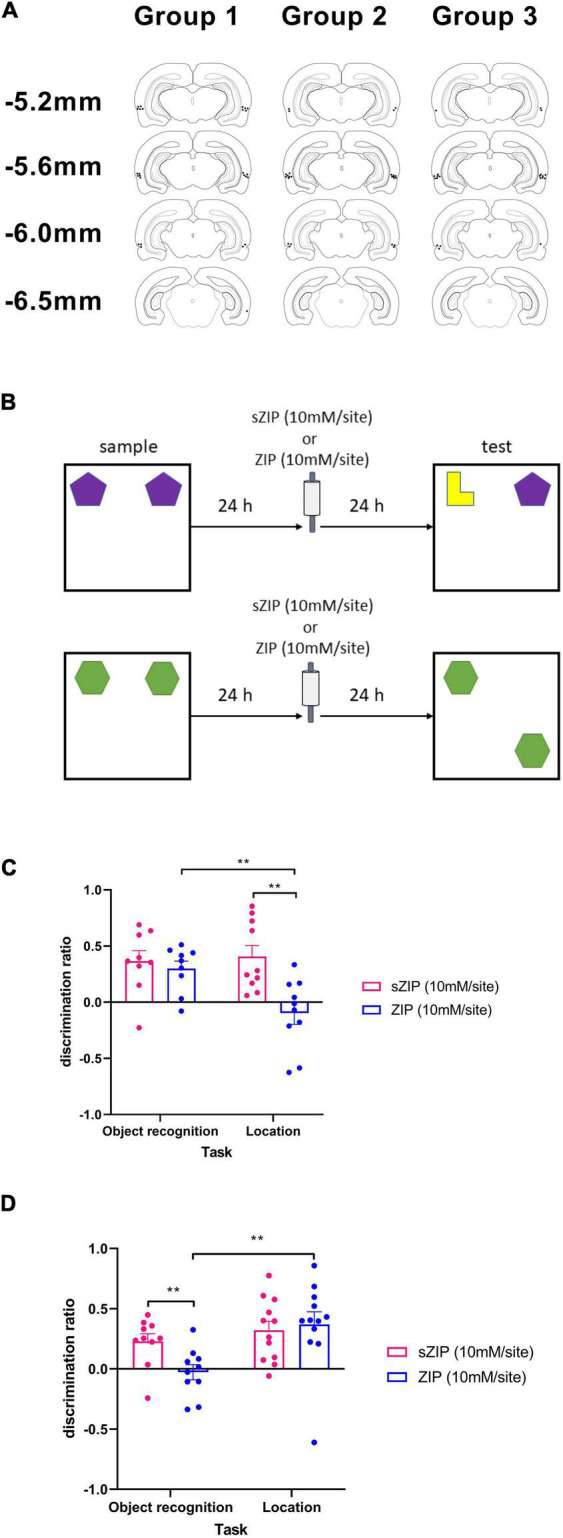
Infusion of ZIP into PRH impaired the maintenance of object recognition but not object location memory. **(A)** Location of cannulae tips targeting PRH in the three groups of animals used in this study. **(B)** Outline of novel object recognition and object location tasks used, ZIP was infused 24 h after the sample phase and 24 h before the test phase. **(C)** Performance in the object location but not the object recognition task was significantly impaired following infusion of ZIP into the HPC. **(D)** Performance in the object recognition task but not the object location task was significantly impaired following infusion of ZIP into PRH. Data presented as mean + sem, **p < 0.01, HPC: NOR *n* = 9, OL *n* = 10, PRH: NOR *n* = 10, OL *n* = 12.

To test the effects of ZIP on memory maintenance, ZIP or sZIP was infused 24 h after the sample phase, and memory was tested at a delay of 48 h (Figure 1B). This timing has been used previously (Pastalkova et al., 2006; Migues et al., 2010) as it allows information to be encoded and for memory to undergo consolidation before ZIP infusion and allows sufficient time after ZIP infusion for memory retrieval not to be affected by the infusion procedure. GluR2_3y_ was infused 1 h before ZIP infusion as this timing has previously been demonstrated to prevent ZIP induced amnesia (Migues et al., 2010). To test the effects of acute ZIP infusion on PRH function in the perceptual oddity task ZIP was infused 15 min before the task. This delay between infusion and behavior is routinely used to test drug effects on PRH function [for example see Barker et al. (2006) and Barker and Warburton (2008, 2015)]. Experiments were performed using a within-subject cross-over design, thus each animal received both a drug and vehicle infusion in each experiment, with a minimum 48 h gap between each infusion.

### Histology

At the completion of the study each rat was anesthetized with Euthetal (Rhone Merieux, Lyon, France) and perfused transcardially with phosphate buffered saline followed by 4% paraformaldehyde. Following removal, the brain was postfixed in paraformaldehyde for a minimum of 2 h then transferred to 30% sucrose in 0.2 M phosphate buffer for 48 h. Coronal sections (50 μm) were cut on a cryostat and stained with cresyl violet. Cannulae locations were checked against a rat brain atlas (Swanson, 1998).

### Behavioral testing

#### Apparatus

Exploration occurred in an open-topped arena 1 m^2^ made of wood, with sawdust on the floor. The walls inside the arena were surrounded with a black cloth to a height of 1.5 m to obscure external visual stimuli (the black cloth was removed for the object location task). An overhead camera and a video recorder recorded the animal’s behavior for subsequent analysis. The stimuli presented were copies of objects composed of “Duplo” (Lego United Kingdom, Slough, United Kingdom) that varied in shape, color, and size and were too heavy for the animal to displace.

#### Pretraining

After being handled for 1 week, the animals were habituated to the empty arena for 5 min daily for 4 days before the commencement of the behavioral testing.

#### Single item novel object recognition memory

The NOR task (Figure 1A) comprised a sample phase, followed by an object preference test after a delay of 48 h. In the sample phase, duplicate copies of an object were placed near the two corners at either end of one side of the arena (15 cm from each adjacent wall). The animal was placed into the arena facing the center of the opposite wall and allowed a total of either 40 s of object exploration or 4 min in the arena. At test (3 min duration), the animal was replaced in the arena, presented with two objects in the same positions: one object was a third copy of the set of the objects used in the sample phase, and the other object was a novel object. The positions of the objects in the test and the objects used as novel or familiar were counterbalanced between the animals.

#### Object location task

This task comprised a sample phase and a test phase separated by a 48 h delay (Figure 1B). In the sample phase (4 min duration), the subjects were presented with two identical objects placed near the two corners at either end of one side of the arena and the amount of exploration of each object was recorded by the experimenter. In the test phase (3 min duration) another identical copy of the object was placed in the same position as during the sample phase, while a fourth identical object was placed in a novel location. The position of the moved object was counterbalanced between rats.

#### Simultaneous oddity discrimination task

**FIGURE 2:**
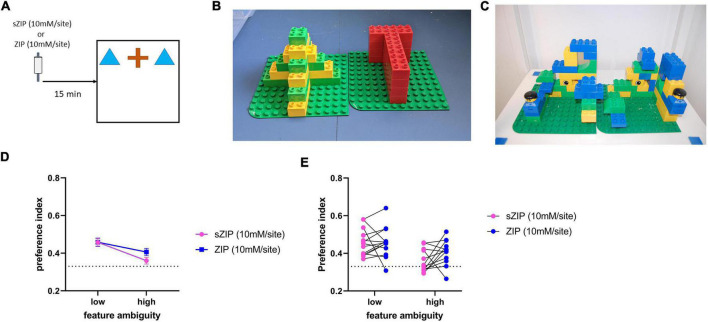
Performance in the perceptual oddity task is not altered by ZIP infusion into the perirhinal cortex. **(A)** Outline of the perceptual oddity task used, ZIP was infused into PRH 15 min before the task commenced. **(B)** Example of low and **(C)** high feature ambiguity objects used in the perceptual oddity task. **(D)** Performance in the perceptual oddity task was not altered in either the low or high feature ambiguity condition by infusion of ZIP, dotted line indicates chance performance levels, data presented as mean ± sem. **(E)** Performance of each individual animal after infusion of sZIP or ZIP on both high and low feature ambiguity conditions. Dotted line represents chance performance (0.33). Low feature ambiguity *n* = 13 for both sZIP & ZIP, high feature ambiguity *n* = 12 for both sZIP and ZIP.

In the perceptual oddity task three objects were presented to the rat simultaneously in a line in the center of the arena (Figure 2A). Two objects were identical, while one object was visually different. Each subject was allowed to explore these three objects for a total of 5 min. In a rat where perception is unimpaired, it has been observed that the animal will spend more time exploring the different object compared to the two identical objects (Bartko et al., 2007). This task was first carried out using a pair of objects with low feature ambiguity. Low feature ambiguity objects are pairs of objects that have few visually overlapping features and are therefore considered less perceptually challenging for the rat to discriminate between (Figure 2B). The task was made more perceptually difficult by repeating it with a pair of objects that had greater feature overlap (Figure 2C). The PRH has been shown to be critical in perceptual discrimination when the stimuli to be discriminated have a high degree of feature overlap (Bussey et al., 2002; Bartko et al., 2007), therefore if ZIP infusion is disrupting PRH function animals’ performance will be impaired in the high feature ambiguity condition.

### Data analysis

All measures of exploration were made with the experimenter blind to the drug status of each animal. Exploratory behavior was defined as the animal directing its nose toward the object at a distance of <2 cm. Any other behavior, such as looking around while sitting on or resting against the object, was not considered as exploration. Discrimination between the objects was calculated using a discrimination ratio (DR), calculated as the absolute difference in the time spent exploring the novel and familiar objects divided by the total time spent exploring the objects. In the simultaneous oddity discrimination task a preference index was calculated as the time spent exploring the ‘different’ divided by the time spent exploring all three objects. Group comparisons used ANOVA and additional analyses examined whether individual groups had discriminated between the objects, using a one-sample *t*-test (two-tailed) against chance performance (0 for object recognition and location, 0.33 for the oddity discrimination task). All statistical analyses used a significance level of 0.05.

## Results

### Histology

Histological examination the PRH group confirmed that the cannulae tips were located in the PRH between AP −5.2 mm and AP −6.3 mm relative to bregma (Figure 1A) and in the HPC group all cannulae tips were located in the HPC between the dorsal CA1 and CA3 subfields [see Figure 2A and Barker and Warburton (2015) for cannula locations].

### Infusion of zeta inhibitory pseudosubstrate into hippocampus selectively impaired the maintenance of object location memory while infusion of zeta inhibitory pseudosubstrate into perirhinal cortex selectively impaired the maintenance of novel object recognition memory

To examine the effect of ZIP infusion into PRH and HPC on the maintenance of object recognition and object location memory, ZIP (10 mM/site) or the scrambled inactive version of the peptide [sZIP (10 mM/site)] was infused 24 h after the sample phase during a 48 h delay between the sample and test phase (Figure 1B).

Intra-HPC ZIP significantly impaired object location (OL) performance but had no effect on NOR (Figure 1C). Thus, a two-way ANOVA with task and treatment as factors revealed a significant interaction [*F*_(1,17)_ = 5.04, *p* = 0.038] and a significant main effect of treatment [*F*_(1,17)_ = 8.56, *p* = 0.009], but no significant main effect of task [*F*_(1,17)_ = 4.34, *p* = 0.053]. Analysis of the simple main effects revealed that the performance of ZIP infused animals was significantly poorer than the performance of sZIP infused animals in the OL task (*p* = 0.008) and performance in the ZIP infused animals was significantly different between the NOR and OL tasks (*p* = 0.005). Performance of the sZIP infused animals was not significantly different between the two tasks (*p* = 0.767). Further analysis revealed that in the NOR task both sZIP [*t*_(8)_ = 3.90, *p* = 0.005] and ZIP [*t*_(8)_ = 4.432, *p* = 0.002] infused animals showed significant discrimination between the novel and the familiar object, in contrast in OL, sZIP [*t*_(9)_ = 4.16, *p* = 0.002] but not ZIP [*t*_(9)_ = −0.99, *p* = 0.350] infused animals showed significant discrimination between the moved and unmoved objects.

Intra-PRH infusion of ZIP significantly impaired NOR performance but had no effect on OL performance (Figure 1D). A two-way ANOVA with treatment and task as factors revealed a significant interaction [*F*_(1,20)_ = 5.27, *p* = 0.033] and a significant main effect of task [*F*_(1,20)_ = 6.82, *p* = 0.017] but no significant main effect of treatment [*F*_(1,20)_ = 2.41, *p* = 0.136]. Analysis of the simple main effects revealed that the performance of ZIP-infused animals was significantly poorer than sZIP-infused animals in the NOR (*p* = 0.004) and the performance of the ZIP infused animals was significantly poorer in the NOR task compared to the OL task (*p* = 0.006). There was no significant difference in the performance of the sZIP infused animals between the two tasks (p = 0.368). Further analysis revealed that in the OL task both sZIP [*t*_(11)_ = 4.29, *p* = 0.001] and ZIP [*t*_(11)_ = 3.56, *p* = 0.004] infused animals showed significant discrimination between the moved and unmoved object, in contrast in the NOR task sZIP [*t*_(9)_ = 3.61, *p* = 0.006] but not ZIP [*t*_(9)_ = −0.43, *p* = 0.674] infused animals showed significant discrimination between the novel and familiar object.

There was no significant effect of intra-PRH, or intra-HPC ZIP infusion on overall object exploration levels (Table 1). Analysis of total object exploration in the sample phase revealed no significant interaction between treatment and task with infusion into either the HPC [*F*_(1,17)_ = 1.82, *p* = 0.195] or PRH [*F*_(1,20)_ = 1.01, *p* = 0.327] and no significant main effect of treatment [HPC *F*_(1,17)_ = 0.28, *p* = 0.606; PRH *F*_(1,20)_ = 0.01, *p* = 0.921], however there was a significant main effect of task with infusion into either region [HPC *F*_(1,17)_ = 4.78, *p* = 0.043; PRH *F*_(1,20)_ = 1.01, *p* = 0.327] which reflected a greater level of overall object exploration in the sample phase of the OL task in both sZIP and ZIP infused animals (Table 1). Analysis of the total object exploration in the test phase revealed no significant interaction between treatment and task following infusion of ZIP into the HPC [*F*_(1,17)_ = 0.25, *p* = 0.621] or PRH [*F*_(1,20)_ = 0.55, *p* = 0.812] and no significant main effect of treatment [HPC *F*_(1,17)_ = 0.04, *p* = 0.852; PRH *F*_(1,20)_ = 0.55, *p* = 0.466]. There was a significant main effect of task following infusion into the HPC [F_(1,17)_ = 6.48, *p* = 0.021] but not following infusion into the PRH [*F*_(1,20)_ = 0.52, *p* = 0.480].

**TABLE 1:** Mean exploration times in the sample and test phases across all three tasks tested.

Figures	Infusion site	Task	Infusate	Exploration in sample phase (s)	Exploration in test phase (s)
Figure 1C	HPC	NOR.	sZIP	32.1 ± 2.1	23.3 ± 4.2
			ZIP	28.6 ± 2.5	21.8 ± 1.6
		OL.	sZIP	33.3 ± 4.3	16.9 ± 1.5
			ZIP	41.4 ± 4.3	18.4 ± 2.1
Figure 1D	PRH	NOR.	sZIP	22.7 ± 2.0	18.3 ± 1.5
			ZIP	23.3 ± 2.4	19.8 ± 2.0
		OL.	sZIP	33.1 ± 3.7	17.7 ± 1.6
			ZIP	32.3 ± 2.3	18.5 ± 0.9
Figure 2D	PRH	PO low FA	sZIP	n/a	40.7 ± 3.3
			ZIP	n/a	41.9 ± 3.9
		PO high FA	sZIP	n/a	44.1 ± 3.4
			ZIP	n/a	45.6 ± 3.2
Figure 3B	PRH	NOR	sGluA2_3y_	26.2 ± 1.5	21.5 ± 2.2
			GluA2_3y_	25.7 ± 2.2	24.6 ± 1.6
Figure 3C	PRH	NOR	sGluA2_3y_ & ZIP	26.1 ± 2.7	25.1 ± 3.3
			GluA2_3y_ & ZIP	27.4 ± 2.4	24.6 ± 2.4

HPC, hippocampus; PRH, perirhinal cortex; NOR, novel object recognition task; OL, object location task; PO, perceptual oddity; FA, feature ambiguity; Data presented as mean ± sem.

**FIGURE 3:**
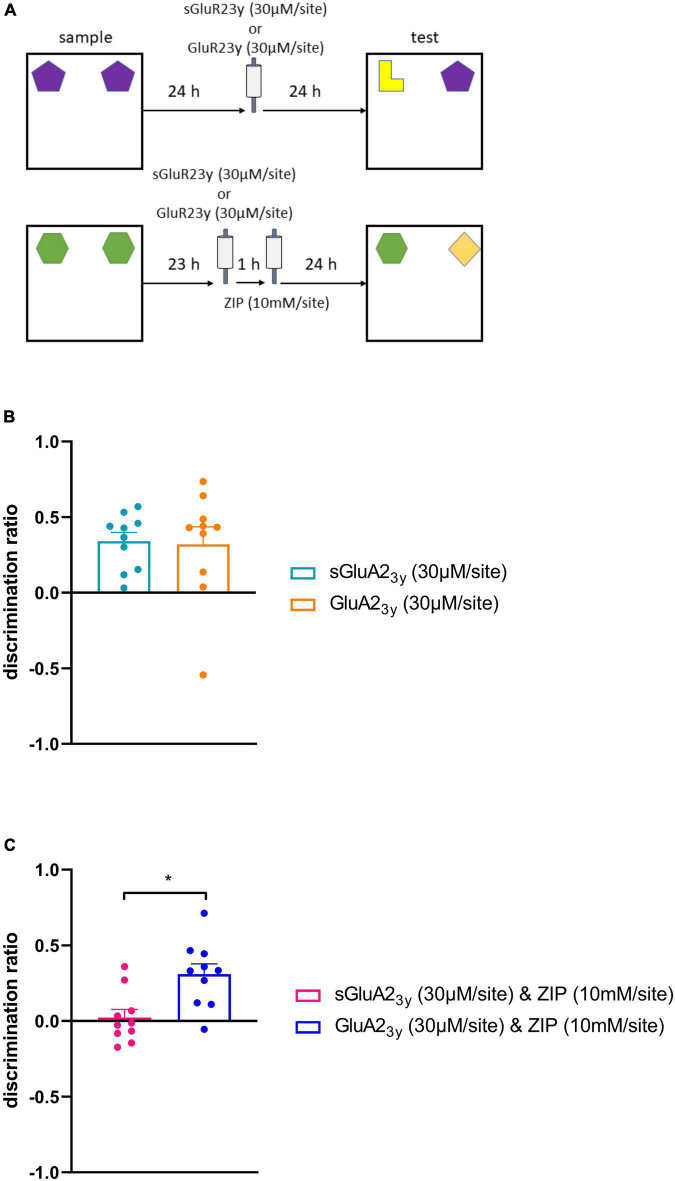
Blocking AMPA receptor endocytosis prevents the impairment in object recognition memory maintenance by intra PRH infusion of ZIP. **(A)** Outline of object recognition task and infusion timings, in the first experiment sGluA23y or GluA23y was infused 24 h after the sample phase, in the second experiment infusion of sGluA23y/GluA23y occurred 23 h after the sample phase, 1 h after this infusion ZIP was infused. **(B)** Intra-PRH GluA23y did not alter NOR memory maintenance. **(C)** Infusion of GluA23y into PRH prevented the impairment in NOR maintenance produced by intra-PRH ZIP. Data presented as mean ± sem, all conditions *n* = 10, *p < 0.05.

### Infusion of zeta inhibitory pseudosubstrate into perirhinal cortex does not alter perceptual function

To investigate the possibility that ZIP infusion produced deficits in NOR performance by impairing perceptual function, rats were tested in a simultaneous oddity discrimination task (Bartko et al., 2007), with a high feature ambiguity and a low feature ambiguity condition, sZIP or ZIP was infused into PRH 15 min before the task (Figure 2A).

Intra-PRH ZIP did not significantly alter performance in either the low or high feature ambiguity condition. Figures 2D,E confirmed by no significant treatment by feature interaction [*F*_(1,23)_ = 1.64, *p* = 0.214] and no significant main effect of treatment [*F*_(1,23)_ = 1.65, *p* = 0.212]. There was a significant main effect of feature ambiguity [*F*_(1,23)_ = 11.65, *p* = 0.002], due to the poorer performance in the sZIP and ZIP infused animals in the high feature ambiguity condition. Further analysis revealed that in the low feature ambiguity condition both sZIP [*t*_(13)_ = 6.26, *p* = 0.00004] and ZIP [*t*_(13)_ = 5.59, *p* = 0.0001] infused animals showed a significant preference for exploring the different object, while in the high feature ambiguity condition the ZIP infused animals showed significant discrimination [*t*_(11)_ = 3.75, *p* = 0.003] but sZIP infused animals did not [*t*_(11)_ = 1.51, *p* = 0.159]. Analysis of the total object exploration revealed no significant interaction between treatment and feature ambiguity [*F*_(1,23)_ = 0.004, *p* = 0.951] and no significant main effect of treatment [*F*_(1,23)_ = 0.15, *p* = 0.702], however there was a significant main effect of feature ambiguity [*F*_(1,23)_ = 4.78, *p* = 0.039], due to the higher levels of exploration completed by both sZIP and ZIP infused animals in the high feature ambiguity condition (Table 1).

### Blocking GluA2 receptor endocytosis prevents the novel object recognition impairment caused by the infusion of zeta inhibitory pseudosubstrate into perirhinal cortex

Infusion of GluA2_3y_ 24 h after the sample phase during a 48 h delay between the sample and test (Figure 3A) phases did not significantly alter performance (Figure 3B). Thus, one-way ANOVA revealed no significant difference between the sGluA2_3y_ and the GluA2_3y_ infused animals [*F*_(1,9)_ = 0.02, *p* = 0.880]. Indeed, animals infused with either sGluA2_3y_ [*t*_(9)_ = 5.87, *p* = 0.0002] or GluA2_3y_ [*t*_(9)_ = 2.75, *p* = 0.022] showed significant discrimination between the novel and familiar objects. In addition, analysis of total object exploration in the sample [*F*_(1,9)_ = 0.03, *p* = 0.872] and test phases [*F*_(1,9)_ = 1.60, *p* = 0.238] revealed no significant difference between the sGluA2_3y_ and GluA2_3y_ infused animals (Table 1).

Infusion of GluA2_3y_ 1 h before ZIP prevented the ZIP induced impairment in performance in the NOR task (Figure 3C). Animals infused with sGluA2_3y_ followed by ZIP showed significantly worse performance in the test phase than animals infused with GluA2_3y_ before ZIP. One-way ANOVA revealed a significant main effect of treatment [*F*_(1,9)_ = 6.66, *p* = 0.03]. Further analysis revealed that animals infused with GluA2_3y_, and ZIP showed significant discrimination between the novel and familiar object [*t*_(9)_ = 4.53, *p* = 0.001], but animals infused with sGluA2_3y_ and ZIP failed to show significant discrimination [*t*_(9)_ = 0.42, *p* = 0.687]. Analysis of the total object exploration in the sample [*F*_(1,9)_ = 0.20, *p* = 0.666] and test phases [*F*_(1,9)_ = 0.02, *p* = 0.903] revealed no significant difference between the sGluA2_3y_/ZIP and GluA2_3y_/ZIP infused animals (Table 1).

## Discussion

The findings of the current study are fourfold: (1) Infusion of ZIP into PRH impaired the maintenance of NOR but not OL memory (2) Infusion of ZIP into HPC impaired the maintenance of OL but not NOR, (3) ZIP infusion did not alter PRH dependent visual-tactile perception, (4) The action of ZIP in blocking memory maintenance is prevented when AMPA receptor endocytosis is blocked by GluA2_3y_.

Administration of ZIP into the HPC and PRH produced impairments in distinct forms of memory, replicating reports of double dissociations in the effects of HPC and PRH lesions (Winters et al., 2004; Barker and Warburton, 2011). The impairment in OL memory following ZIP infusion into HPC is in line with the well-established role of HPC in spatial memory and replicates previous studies (Hardt et al., 2010; Migues et al., 2010). The role of the HPC in object recognition memory is complex, as some studies fail to report NOR deficits following lesion of the HPC [see Brown and Banks (2015); Cohen and Stackman (2015), and Chao et al. (2020) for reviews], while others have reported NOR deficits specifically following HPC drug infusions [see Cohen and Stackman (2015) and Chao et al. (2020) for reviews]. Here, the failure of intra dorsal HPC ZIP to alter NOR performance replicates a previous study (Hardt et al., 2010). In contrast another study reported that ZIP infusion into the dorsal, intermediate and ventral hippocampus, NOR performance was significantly impaired compared to controls (Hales et al., 2015) although it should be noted that the ZIP-treated animals were still able to discriminate between the novel and familiar objects. Thus, it appears that while the HPC as a whole may play a role in the maintenance of NOR memory, the dorsal HPC alone does not. In addition, any role of the HPC is not as critical as that of the PRH. That intra-PRH ZIP selectively impaired NOR demonstrates that object recognition memory information is stored in the PRH for at least 24 h, in line with findings from *in vivo* recording studies (Xiang and Brown, 1998) and this finding is replicated by a further study in this issue (Augereau and Hardt) which extends the finding to show that 6 days old object memories are also dependent on PRH.

ZIP has been shown to impair the maintenance of LTP but not LTD *in vitro* (Sajikumar et al., 2005; Panaccione et al., 2013), and it has been hypothesized that PKMζ maintains memories by preventing GluA2 receptor endocytosis (Sacktor, 2011), In this study ZIP induced amnesia was prevented by blocking GluA2 receptor endocytosis, using the synthetic peptide GluA2_3y_ indicating that ZIP impairs the maintenance of memory and LTP by the same mechanism. Thus, this study provides evidence that LTP-like in addition to LTD-like mechanisms within PRH are critical for object recognition memory.

Previous reports have failed to find a clear link between LTP and object recognition memory formation within PRH. Thus, blockade of cannabinoid or NR2A receptors which was found to impair LTP, but not LTD produced no impairment in NOR when infused into the PRH (Massey et al., 2004; Barker et al., 2006; Tamagnini et al., 2013). Although some studies have suggested a correlational link between LTP and PRH dependent object memory (Silingardi et al., 2011), The discrepancy in these findings might reflect the involvement of different forms of LTP, or that different forms of plasticity mediate different stages of memory processing (i.e., memory encoding vs. maintenance).

Although a number of studies have demonstrated a link between LTD-like mechanisms in PRH and object recognition memory (Warburton et al., 2003; Griffiths et al., 2008), the observation that LTP-like processes also pay a role is not unexpected. Mathematical models have demonstrated the importance of strengthening some synapses while others are weakened for efficient network function (Norman, 2010) and if synaptic weakening was the only process occurring within PRH then object recognition memory capacity would be highly limited as synaptic weakening alone would lead to a loss of neuronal responses within PRH. However, investigation of human recognition memory revealed subjects were able to remember 10,000 images with the same accuracy as 100 (Standing, 1973), suggesting that humans have a large capacity for recognition. Understanding the relationship between LTD and LTP-like processes during object recognition memory formation and maintenance will be critical to understanding how PRH is able to support the large capacity of object recognition memory.

Infusion of ZIP into either the HPC or PRH did not alter the animals overall object exploration levels in any of the tasks tested in this study, indicating that ZIP did not alter the animals motivation to interact with the stimuli or alter attentional processing. In experiment 1 exploration levels during the sample phase were higher in all conditions during OL compared to NOR, as object exploration in NOR was capped at 40 s, whereas, there was no limit on object exploration in the OL. Given we observed a double dissociation in the effects of HPC and PRH ZIP function on performance in these tasks it is unlikely that this difference in overall exploration levels between the tasks contributed any of the observed deficits.

Zeta inhibitory pseudosubstrate is thought to act by blocking the catalytic domain of atypical PKCs, however a recent report found that infusion of ZIP into the HPC impaired synaptic transmission (LeBlancq et al., 2016). Therefore, to test whether the infusion of ZIP into PRH had a non-specific effect we assessed performance in an oddity discrimination task, in which, when objects have a high degree of feature overlap is sensitive to disruption of synaptic transmission in the PRH (Bussey et al., 2002; Bartko et al., 2007). Here, infusion of ZIP into PRH did not disrupt performance in the perceptual oddity discrimination task, suggesting that ZIP infusion did not disrupt synaptic transmission within PRH. It has been reported that administration of ZIP onto cultured hippocampal neurons can result in cell death (Sadeh et al., 2015), however in the present experiments a within subjects design was used, and animals also received multiple infusions yet no change in behavioral performance was observed following ZIP infusion suggesting that ZIP has not caused large scale cell death. Other studies have also shown that animals can form new memories following ZIP infusion further suggesting that the effect of ZIP is not due to cell death (Pastalkova et al., 2006; Sacktor, 2008; von Kraus et al., 2010; Hales et al., 2015). Therefore, it is unlikely that the observed deficits in performance were due to cell death.

In summary, this study demonstrated that OR memory is maintained in the PRH not the HPC and demonstrated the importance of LTP-like mechanisms within PRH to the maintenance of object recognition memory. Understanding how LTD and LTP like processes interact within PRH will be critical to understanding object recognition memory formation and maintenance.

## Data availability statement

The raw data supporting the conclusions of this article will be made available by the authors, without undue reservation.

## Ethics statement

This animal study was reviewed and approved by Animal Welfare and Ethical Review Body (AWERB), University of Bristol.

## Author contributions

AO and GB performed the experiments and analyzed the data. All authors designed the experiments, wrote the manuscript, and approved the submitted version.
